# Dietary Acid Load, Empirical Dietary Inflammatory Index, and Literature‐Based Adherence to Mediterranean Diet Score Relationship With Primary Dysmenorrhea

**DOI:** 10.1002/fsn3.71580

**Published:** 2026-04-27

**Authors:** Mina Salek, Farshad Teymoori, Ladan Haghighi, Masoud Salehi, Saeed Tavakkoli, Minoo HasanRashedi, Farzad Shidfar

**Affiliations:** ^1^ Department of Nutrition, School of Public Health Iran University of Medical Sciences Tehran Iran; ^2^ Nutritional Sciences Research Center Iran University of Medical Sciences Tehran Iran; ^3^ Department of Obstetrics and Gynecology, School of Medicine Iran University of Medical Sciences Tehran Iran; ^4^ Department of Biostatistics, School of Public Health Iran University of Medical Sciences Tehran Iran

**Keywords:** dietary acid load, dietary inflammatory index, mediterranean diet, menstrual health, primary dysmenorrhea

## Abstract

Primary dysmenorrhea is a prevalent health issue that has a negative impact on women's well‐being. This study sought to explore the potential connection between dietary indices including dietary acid load (DAL), empirical dietary inflammatory index (eDII), and Literature‐Based Adherence to Mediterranean diet Score (MEDI‐LITE) with primary dysmenorrhea. A total of 105 university students including 57 with and 48 without primary dysmenorrhea participated in this cross‐sectional study. Dietary data were collected using a 117‐time Food Frequency Questionnaire (FFQ), and the dietary indices were calculated using developed formulas. Using pre‐designed questionnaires, the Visual Analog Scale (VAS) and the Verbal Multidimensional Scoring System (VMS) were computed and used to determine the severity of dysmenorrhea pain and side effects. Dietary indices and primary dysmenorrhea relationship assessed by regression analysis adjusted for potential confounders. In the adjusted linear regression models, none of the dietary indices, including eDII, MEDI‐LITE, and DAL, showed a statistically significant association with VAS scores. Similarly, the odds of primary dysmenorrhea across tertiles of these dietary indices did not differ significantly. No significant associations were observed between any of the dietary indices and the severity or complications of dysmenorrhea. The result of ROC analysis showed that the mentioned dietary indices were not significant predictors of dysmenorrhea status. The findings of our study showed no significant association between DAL, eDII, and MEDI‐LITE and primary dysmenorrhea. Further, well‐designed prospective studies are required to confirm these findings, with particular attention to the timing and pattern of dysmenorrhea symptoms.

## Graphical abstract

This cross‐sectional study investigated the associations between dietary acid load (DAL), empirical dietary inflammatory index (eDII), and adherence to the Mediterranean diet (MEDI‐LITE) with primary dysmenorrhea among 105 university students. Dietary intake was assessed using a validated Food Frequency Questionnaire, and dysmenorrhea severity was measured using VAS and VMS tools, with data analyzed via regression models adjusted for confounders. The results revealed no significant associations between the evaluated dietary indices and the occurrence or severity of primary dysmenorrhea, suggesting the need for further research to confirm these findings.

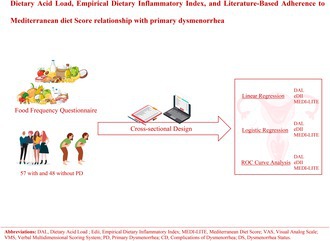

## 1 Introduction

Around three‐quarters of women experience severe lower abdominal pain during their menstrual cycle, known as primary dysmenorrhea (Armour et al. 2019). Dysmenorrhea is a highly prevalent condition among students, with an estimated occurrence of approximately 70%, and its incidence has shown an increasing trend over the past decade. At least half of those affected report symptoms of mild severity (Wang et al. 2022; Liu et al. 2024). This condition can have a negative impact on the quality of life of women, leading to decreased productivity at work, absenteeism from school, and increased use of pain medication, resulting in economic losses (Barcikowska, Rajkowska‐Labon, et al. 2020; Dawood 2006). While gynecological factors, such as menstrual flow characteristics and family history, are recognized contributors, growing evidence highlights the significant role of modifiable lifestyle factors in the onset and exacerbation of dysmenorrhea (Wang et al. 2022). Among these factors, dietary habits represent a central and modifiable component that interacts with other behaviors—such as sleep quality, duration, and mental health, particularly during menstruation. Dietary components, including caffeine intake, consumption of cold or spicy foods, and irregular meal patterns like breakfast skipping, have all been closely associated with the intensity and progression of dysmenorrhea symptoms (Wang et al. 2022; Liu et al. 2024).

Recent research highlights the important role of nutrition in dysmenorrhea, with inflammatory processes, particularly elevated prostaglandin (F2a and E2) secretion during the luteal phase, that can intensify uterine contractions, leading to vasoconstriction, ischemia, and pain (Barcikowska, Rajkowska‐Labon, et al. 2020; Kanauchi et al. 2019; Tabung et al. 2016). Although several studies have explored the impact of individual dietary components on inflammation, both at the food group level (Tadese et al. 2021; Monday et al. 2019; Kartal and Akyuz 2018a; Najafi et al. 2018; Bajalan et al. 2019; Hailemeskel et al. 2016) and at the nutrient level (Fitrianingsih and Santanu 2021; Naz et al. 2020; Abdi et al. 2021; Amirkhizi et al. 2023), limited research has assessed the overall inflammatory or anti‐inflammatory potential of habitual dietary patterns in relation to dysmenorrhea (Najafi et al. 2018; Mucuk and Onur 2021; Onieva‐Zafra et al. 2020). In one study conducted among Turkish university students, higher adherence to the Dietary Inflammatory Index (DII) was not significantly associated with primary dysmenorrhea, possibly due to the homogeneity of participant characteristics across groups (Mucuk and Onur 2021). In contrast, another study found that adherence to an inflammatory dietary pattern, characterized by high intake of salty, caffeinated, and sugar‐rich foods, was associated with an increased risk of dysmenorrhea during menstruation in young women, while adherence to vegetarian or dairy‐based patterns showed no significant association; the potential pro‐inflammatory effects of dairy products may explain this neutral outcome (Najafi et al. 2018). Additionally, lower adherence to the Mediterranean diet has been associated with longer menstrual cycles, though not with menstrual pain or bleeding, while higher intake of fruits has been linked to reduced pain severity, and greater consumption of fish and eggs has been associated with less frequent occurrence of dysmenorrhea (Onieva‐Zafra et al. 2020; Balbi et al. 2000). Moreover, a three‐month intervention using an anti‐inflammatory Mediterranean‐style diet, alongside standard medical treatment, led to reductions in menstrual pain and distress, although the effects were not significantly different from medical treatment alone (Mohamed et al. 2024).

Moreover, studies have shown that women with dysmenorrhea exhibit elevated levels of oxidative stress and metabolic acidosis‐related factors (Mucuk and Onur 2021; Ma et al. 2021), which may contribute to pain perception by promoting inflammatory responses and nerve injury (Wu et al. 2019). A high dietary acid load can further disrupt acid–base homeostasis, leading to mild chronic metabolic acidosis that exacerbates tissue damage and systemic inflammation (Wu et al. 2019; Williams et al. 2016; Jafari et al. 2021). In a previous cross‐sectional study, higher DAL, indicating less pain‐protector nutraceutical, was related to more intense musculoskeletal pain (Bahrampour and Clark 2022).

Given that previous studies have not comprehensively examined the inflammatory potential and acidity of the diet in relation to dysmenorrhea‐related pain, nor adequately assessed adherence to the Mediterranean diet, this study aimed to address these gaps by evaluating three established literature‐based dietary indices: the Empirical Dietary Inflammatory Index (eDII), which includes eight pro‐inflammatory and eight anti‐inflammatory foods and is linked to biomarkers of inflammation (Kanauchi et al. 2019); the Dietary Acid Load (DAL), which reflects the net acid‐producing potential of the diet based on the balance between acidic and alkaline food intake (Scialla and Anderson 2013); and the Literature‐based adherence score to the Mediterranean diet (MEDI‐LITE score), a validated and consistent tool for assessing individual adherence to the Mediterranean diet (Sofi et al. 2014). This study aimed to examine the possible associations between the above‐mentioned dietary indices and the severity of dysmenorrhea‐related pain.

## 2 Material and Methods

### 2.1 Study Participants

The present cross‐sectional study was conducted on female students (with or without dysmenorrhea) of the Iran University of Medical Science (IUMS), Tehran, Iran. The samples were taken by an easy and consecutive sampling method among the available students and the sampling continued until the final sample size was reached.

The sample size was calculated using the standard formula for cross‐sectional studies using the mean and standard deviation of the DII reported in a previous study by Mucuk et al. (Mucuk and Onur 2021) which was 5.01 ± 1.23 and 3.68 ± 1.30 among dysmenorrhea and non‐dysmenorrhea students, considering the 95% confidence interval (alpha = 0.05), the statistical power of 90%, and the probability of a 25% drop in the samples, and we reached a total of 80 samples (40 samples in each group). To increase the study power, it was tried to enter more samples which finally after applying the exclusion criteria to participating students, 57 dysmenorrhea and 48 non‐dysmenorrhea students were included for the final analysis.

The formula for calculating the sample size is as follows:

$$n=\frac{{\left(Z_{1}+Z_{2}\right)}^{2}\left(s_{1}^{2}+s_{2}^{2}\right)}{{\left(\mu_{1}-\mu_{2}\right)}^{2}}$$

### 2.2 Inclusion and Exclusion Criteria

Participants were required to be women IUMS students aged 18 to 25, with a normal BMI (18.5–24.9), willing to participate in the study, and their primary dysmenorrhea status was ascertained by a gynecologist after filling the pre‐designed questionnaire for diagnosis of primary dysmenorrhea, the Visual Analog Scale (VAS), and checking the other pre‐defined questionnaires.

Exclusion criteria consisted of sexually active students, those who were on a special diet, smokers, individuals with pelvic pathology (such as myoma, pelvic tumors, endometriosis, and pelvic infection), any chronic diseases, or those who regularly used medication, supplements, or antibiotics, and a history of any diagnosis of secondary gynecological disorders.

### 2.3 Data Measurements

Pelvic pathology was determined through a questionnaire created by the research team based on previous studies and under the supervision of an obstetrician and gynecologist. The data were collected using a face‐to‐face interview method through a survey form that included questions about socio‐demographic characteristics, obstetric/gynecological histories, daily life activities, the Visual Analog Scale (VAS), and the Verbal Multidimensional Scoring system (VMS) for determining the primary dysmenorrhea status.

A pre‐designed questionnaire was used for collecting the general and demographic information.

Written informed consent was obtained from all participants, and the study protocol was reviewed and approved by the ethics research council of the IUMS by the ethics code of IR.IUMS.REC.1400.213.

### 2.4 Outcome Measurement

Participants were divided into two groups with or without primary dysmenorrhea based on the results of the Visual Analog Scale (VAS) that a person specifies the intensity of his felt pain in a range between 0 and 100 scores which has been shown on a 10‐cm line on a page (Couper et al. 2006). Scores higher than 4 were ascertained as primary dysmenorrhea. Scores between 5 and 44, 45 and 74, and 75 and 100 are defined as mild, moderate, and severe pain, respectively. Also, the Verbal Multidimensional Scoring System (VMS) was used, which is a validated tool for assessing the severity of primary dysmenorrhea and the impact on daily activities (Shah et al. 2016). It classifies dysmenorrhea into four scores: score 0 indicates no pain or interference with activities; score 1 reflects mild pain with minimal disruption and infrequent need for analgesics; score 2 denotes moderate pain that affects daily functioning but responds well to medication; and score 3 represents severe pain with significant activity limitation, poor response to analgesics, and the presence of vegetative symptoms such as headache, fatigue, or gastrointestinal distress.

### 2.5 Physical Activity

Physical activity was measured using the validated version of the Baecke questionnaire among the Iranian population (Sadeghisani et al. 2016). The Baecke questionnaire consists of 16 questions within three main domains at the level of individual physical activities (occupational, sport, and recreational) that evaluate the habitual physical activities in the previous 12 months (Baecke et al. 1982). Total physical activity is achieved by calculating the sum of the scores obtained from occupational, sport, and recreational categories.

### 2.6 Dietary Measurement

During a face‐to‐face interview with trained dietitians, the usual food intakes during the previous year were obtained using a valid and reliable Semi‐Quantitative Food Frequency Questionnaire (FFQ) with 117 items for Iranian foods (Malekshah et al. 2006). Consumption amounts of each food item using conventional and standard portion sizes were questioned. Based on this questionnaire, each person was asked to report his/her dietary intake of each food item listed in the questionnaire based on a 9‐option answer grouping (from never or less than once per month to 6 times or more per day). Portion sizes of food frequencies are entered into an Excel sheet and then converted into a gram scale. Daily energy and nutrient intake, particularly protein, phosphorus, potassium, magnesium, and calcium, were computed through Nutritionist IV software using the United States Department of Agriculture's (USDA) Food Composition Table (FCT) and also Iranian FCT for some local foods that were not listed in USDA FCT (Azar and Sarkisian 1980).

### 2.7 Exposure Assessment

#### 2.7.1 eDII

This index was computed using the method developed by Kanauchi et al. (Kanauchi et al. 2019) using 8 pro‐inflammatory (red meats, processed meats, organ meats, other fish, eggs, sugar‐sweetened beverages, tomatoes, and refined grains) and 8 anti‐inflammatory components (leafy green vegetables, dark yellow vegetables, fruit juice, oily fish, coffee, tea, wine, and beer or other alcohol beverages). A high, moderate, and low consumption of pro‐inflammatory food groups based on the serving number cut point determined by Kanauchi et al. takes the score of +2, +1, and 0, respectively. Whereas, in the same way for anti‐inflammatory components, the scores included as −2, −1, and 0.

The scores for all 16 components were summed and total scores ranged from −16 to +16, with a higher score indicating a higher inflammatory potential. Of note, the eDII includes both pro‐inflammatory and anti‐inflammatory food groups with established links to systemic inflammation, offering a simplified, food‐based approach that reduces reliance on complex nutrient databases and minimizes potential errors associated with nutrient estimation.

#### 2.7.2 MEDI‐LITE

MEDI‐LITE index was calculated by the developed method by Sofi et al. (Sofi et al. 2014) and it consists of nine different food categories: fruit, vegetables, grains, legumes, fish and fish products, meat and meat products, dairy products, alcohol, and olive oil. Each food group that is traditionally part of the Mediterranean diet, such as fruits, vegetables, grains, legumes, and fish, is assigned a score ranging from 2 for highest consumption to 0 for lower consumption based on the proposed serving numbers in the development study. For the meat food group and dairy products, a score of 2 is given for lower consumption, 1 for intermediate consumption, and 0 for higher consumption based on the proposed serving numbers in the development study by Sofi et al. (Sofi et al. 2014).

For olive oil intake, 2 points were assigned to daily intake, 1 point to frequent use, and 0 for occasional consumption. Due to cultural and religious reasons, alcohol consumption is very rare, especially among Iranian women, and on the other hand, consumers usually avoid reporting their consumption, so the alcohol consumption was not recorded and we excluded it in the Medi‐Lite scoring. Consequently, the highest possible Medi‐Lite score was 16 instead of 18.

A higher MEDI‐LITE score indicated higher adherence to the Mediterranean diet.

#### 2.7.3 DAL

DAL index was calculated using this developed formula: DAL (mEq/day) = PRAL + [(body surface area (m^2^) × 41 (mEq/day)) /1/73 m^2^]. The potential renal acid load (PRAL) and Body Surface Area (BSA) were calculated as follows: PRAL (mEq/d) = (0.49 × protein (g/d)) + (0.037 × phosphorus (mg/d)) − (0.021 × potassium (mg/d)) − (0.026 × magnesium (mg/d)) − (0.013 × calcium (mg/d)). BSA (m^2^) = 0/007184 × height (cm)^0/725^ × weight (kg)^0/425^ (Scialla and Anderson 2013).

A higher DAL score indicated a higher acidic potential of the diet.

### 2.8 Statistical Analysis

Data analysis was conducted using the Statistical Package for Social Sciences software (SPSS) version 25. The normality of data was assessed using the histogram charts and Kolmogorov–Smirnov analysis. Population characteristics for continuous variables and categorical variables are presented as mean ± standard deviation (SD) and number (percentages), respectively. Comparing the mean and distribution of variables between students with and without dysmenorrhea was conducted using independent sample *t*‐test and chi‐square test for quantitative and qualitative variables, respectively.

The correlation coefficient (*r*) between dietary indices and VAS score was calculated using the Pearson correlation test. Linear regression test was used to assess the association of each unit increment of dietary indices and VAS score, adjusted for potential literature‐based confounders including age, menarche age, education levels, physical activity, BMI, and energy intake. To examine the association between adherence to dietary indices and dysmenorrhea‐related outcomes, we categorized each dietary index into tertiles representing low, moderate, and high adherence levels. Logistic regression analysis was performed in both crude and adjusted models (accounting for relevant confounders) to estimate the odds of primary dysmenorrhea, severe menstrual pain, and severe dysmenorrhea‐related complications across these adherence levels. The lowest tertile served as the reference group, and odds ratios (ORs) for the second and third tertiles were calculated to assess the strength of association. The OR with a 95% confidence interval (CI) was reported and *p*‐values < 0.05 were considered statistically significant. To complement the regression analyses, we assessed the discriminative ability of dietary indices for differentiating dysmenorrhea status using ROC curve analysis. The area under the curve (AUC) was calculated to assess the accuracy of the model, with models having an AUC greater than 0.7 considered to have acceptable accuracy. In addition, cut‐off points corresponding to complete sensitivity and specificity were identified where applicable.

## 3 Results

Population characteristics of study participants are presented in Table 1. Subjects with dysmenorrhea had significantly higher scores of VAS, VMS, and dietary intake of carbohydrates, fiber, and vitamin C compared with those with non‐dysmenorrhea (*p*‐value < 0.05). There were no significant differences between the two groups in participants' age, menarche age, BMI, physical activity, education levels, and dietary intake of energy, protein, fat, vitamin E, calcium, and dietary indices including DAL, eDII, and MEDI‐LITE (All *p*‐values > 0.05).

**TABLE 1:** Population characteristics of study participants.

Characteristic	Group		*p*
	Dysmenorrhea (*n* = 57)	Non‐dysmenorrhea (*n* = 48)	
Age (year)	22.6 ± 2.12	23.0 ± 2.06	0.301
Menarche age (year)	12.8 ± 1.31	13.0 ± 1.25	0.463
BMI (kg/m^2^)	21.6 ± 1.91	22.0 ± 1.83	0.268
Physical activity	7.5 ± 1.14	7.4 ± 0.87	0.335
Education levels			0.200
Undergraduate student (%)	80.0	71.4	
Master's degree student and above (%)	20.0	28.2	
VAS	71.1 ± 11.99	1.6 ± 1.27	< 0.001
VMS			< 0.001
Grade 0	—	48 (100)	
Mild (Grade 1)	7 (12.3)	—	
Moderate (Grade 2)	37 (64.9)	—	
**Dietary intakes**			
Energy (Kcal/d)	1968 ± 482	2135 ± 503	0.087
Carbohydrate (g/d)	375 ± 177	309 ± 132	0.035
Protein (g/d)	88.3 ± 36.0	80.0 ± 37.0	0.242
Fat (g/d)	89.2 ± 23.4	55.3 ± 25.1	0.415
Fiber (g/d)	22.3 ± 12.3	15.7 ± 6.1	0.001
Vitamin C (mg/d)	131.5 ± 65.0	71.5 ± 35.6	0.001
Vitamin E (mg/d)	19.7 ± 7.1	18.9 ± 6.3	0.592
Calcium (mg/d)	857 ± 323	807 ± 327	0.433
**Dietary indices**			
eDII	1.2 ± 1.65	1.4 ± 1.23	0.548
MEDI‐LITE	6.5 ± 1.81	6.9 ± 1.54	0.256
DAL (mEq/d)	37.9 ± 32.0	46.1 ± 28.6	0.174

Abbreviations: BMI, body mass index; DAL, dietary acid loadeDII, empirical dietary inflammatory index; MEDI‐LITE, literature‐based adherence to mediterranean diet Score; VAS, visual analog scale; VMS, verbal multidimensional scoring system.

Table 2 shows the correlation coefficient of dietary indices with VAS score among study participants. The dietary indices were not correlated with VAS and the correlation coefficients for eDII, MEDI‐LITE, and DAL with VAS scores were −0.027, −0.098, and −0.116 respectively.

**TABLE 2:** The correlation of dietary indices with VAS score among study participants.

Dietary indices	Correlation coefficient (r)	*p*
Empirical dietary inflammatory index (eDII)	−0.027	0.792
Literature‐Based adherence to mediterranean diet score (MEDI‐LITE)	−0.098	0.335
Dietary acid load (DAL)	−0.116	0.252

Table 3 presents the findings of linear regression analysis on the association between dietary indices and VAS score. Findings indicated that one unit increment of each dietary index including eDII (β = −0.39, 95% CI (−5.17, 4.39), *p*‐value = 0.871), MEDI‐LITE (β = −1.80, 95% CI (−5.89, 2.29), *p*‐value = 0.385), and DAL (β = −0.13, 95% CI (−0.36, 0.10), *p*‐value = 0.259), were not significantly associated with VAS score in the final adjusted model of analysis.

**TABLE 3:** The beta coefficient and 95% confidence interval of visual analog scale (VAS) per one unit of dietary indices among study participants.

Predictor variables	Regression coefficients			
	Unstandardized *β* (95% CI)	se	Adjusted *R* Square	*p*
**eDII**				
Crude model	−1.36 (−6.12, 3.38)	2.39	0.007	0.570
Adjusted model	−0.39 (−5.17, 4.39)	2.41	0.42	0.871
**MEDI‐LITE**				
Crude model	−2.28 (−6.39, 1.82)	2.07	0.002	0.272
Adjusted model	−1.80 (−5.89, 2.29)	2.06	0.49	0.385
**DAL**				
Crude model	−0.15 (−0.38, 0.07)	0.114	0.008	0.176
Adjusted model	−0.13 (−0.36, 0.10)	0.117	0.54	0.259

*Note:* Analyses were adjusted for age, menarche age, education levels, physical activity, BMI, and energy intake.

Abbreviations: DAL, dietary acid load; eDII, empirical dietary inflammatory index; MEDI‐LITE; literature‐based adherence to mediterranean diet score.

The OR (95% CI) of dysmenorrhea across tertiles of dietary indices is indicated in Table 4. In the crude model of logistic regression analysis, none of the dietary indices were related to dysmenorrhea. In addition, in the adjusted model accounting for potential confounders, no statistically significant associations were observed between dietary indices and dysmenorrhea. The odds ratios (OR) and 95% confidence intervals (CI) for individuals in the highest versus lowest tertile were as follows: for eDII, OR = 1.05 (95% CI: 0.31–3.59; *p* = 0.927); for MEDI‐LITE, OR = 0.69 (95% CI: 0.17–2.73; *p* = 0.598); and for DAL, OR = 1.69 (95% CI: 0.57–4.98; *p* = 0.340).

**TABLE 4:** The odds ratio and 95% confidence interval of primary dysmenorrhea across tertiles of dietary indices among study participants.

Predictor variables	Tertile 1	Tertile 2		Tertile 3	
	Ref	OR (95% CI)	*p*	OR (95% CI)	*p*
**eDII**					
Crude model	**Ref**	1.57 (0.55–4.45)	0.393	1.11 (0.34–3.56)	0.859
Adjusted model	**Ref**	1.35 (0.43–4.20)	0.595	1.05 (0.31–3.59)	0.927
**MEDI‐LITE**					
Crude model	**Ref**	1.88 (0.52–6.73)	0.331	0.76 (0.21–2.73)	0.674
Adjusted model	**Ref**	1.80 (0.45–7.13)	0.401	0.69 (0.17–2.73)	0.598
**DAL (mEq/d)**					
Crude model	**Ref**	1.12 (0.43–2.86)	0.811	1.79 (0.69–4.65)	0.231
Adjusted model	**Ref**	1.03 (0.37–2.86)	0.941	1.69 (0.57–4.98)	0.340

*Note:* Analyses were adjusted for age, menarche age, education levels, physical activity, BMI, and energy intake.

Abbreviations: DAL, dietary acid load; eDII, empirical dietary inflammatory index; MEDI‐LITE, literature‐based adherence to mediterranean diet score.

**FIGURE 1:**
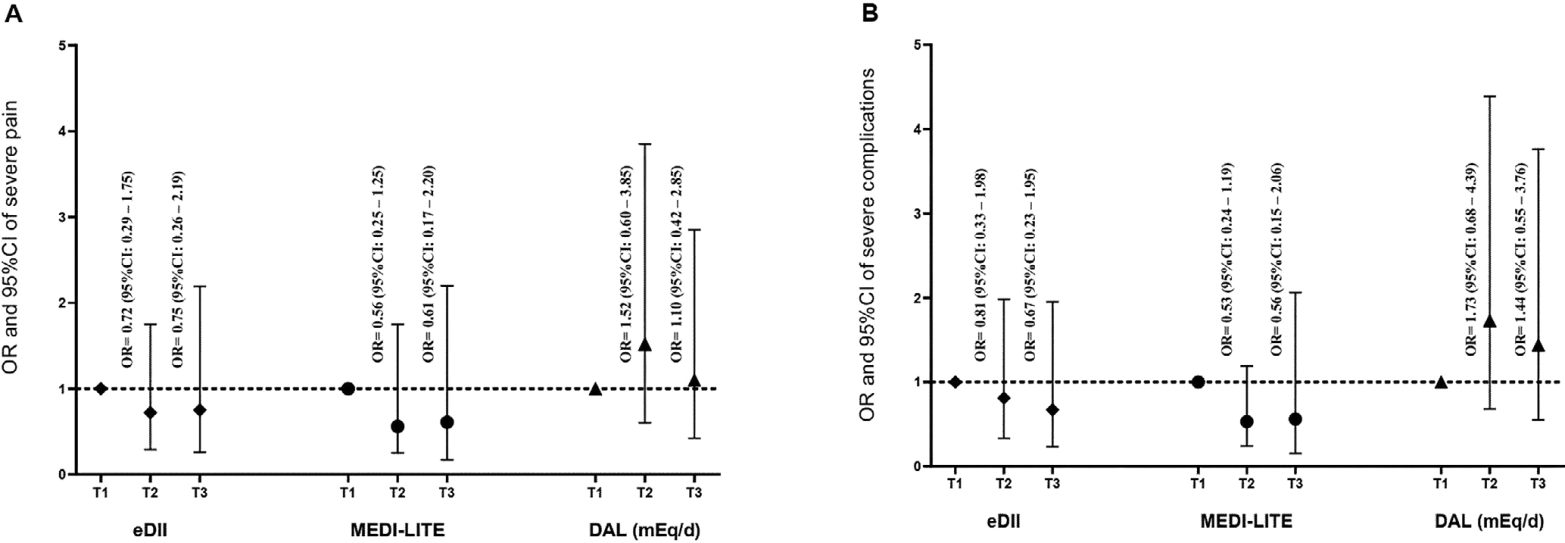
Odds ratio and 95% confidence interval of (A) severe dysmenorrhea pain (based on VAS) and (B) severe dysmenorrhea complications (based on VMS) across tertiles of dietary indices in the adjusted logistic regression model.

**FIGURE 2:**
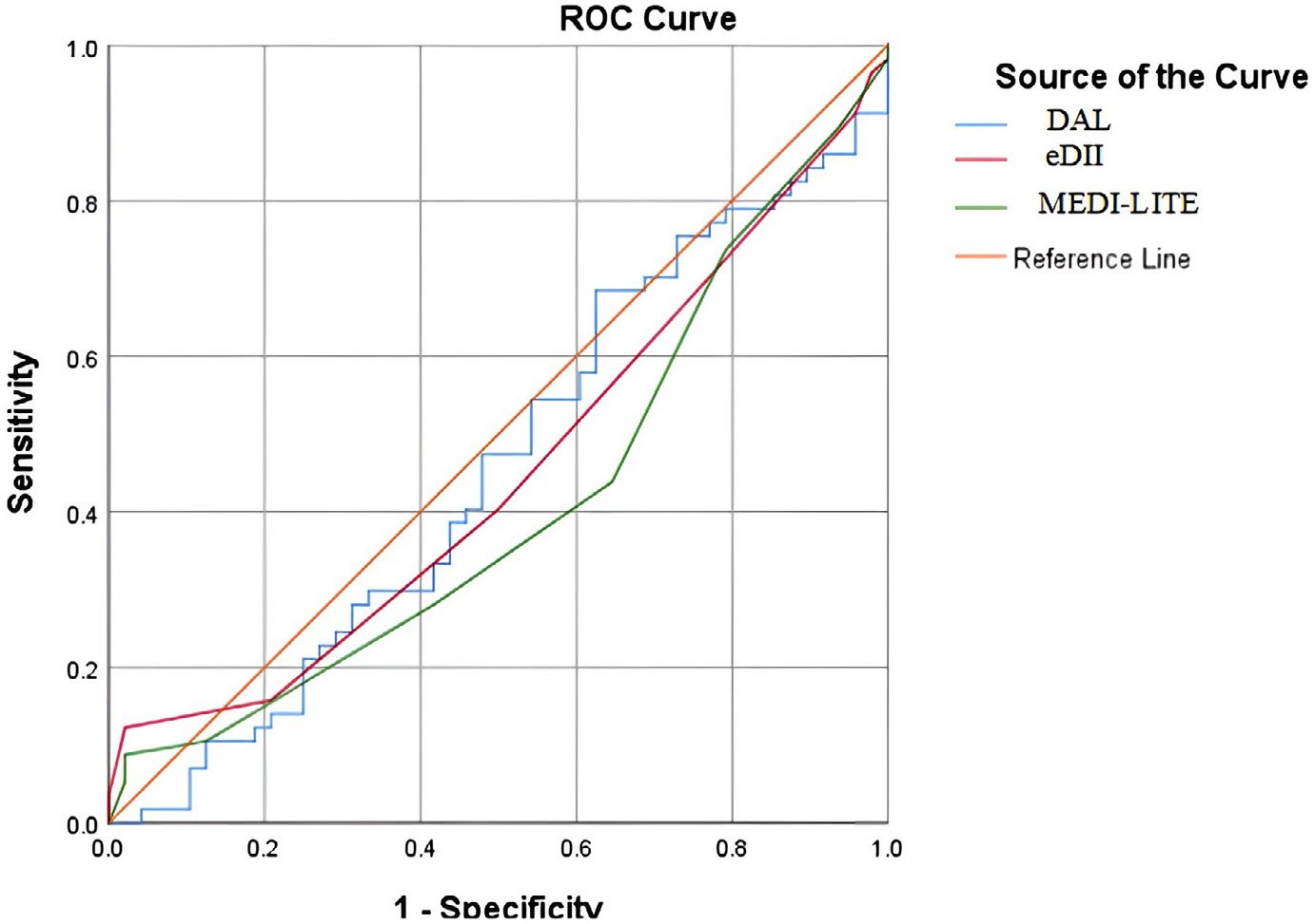
ROC curve analysis for evaluating the sensitivity and specificity of dietary indices in differentiating dysmenorrhea status. The reference line represents a random classifier (AUC = 0.5) and serves as a visual indicator of significance; curves approaching or below this line suggest non‐significant predictive ability.

The adjusted model of dietary indices relationship with the odds of severe pain of dysmenorrhea and severe complications of dysmenorrhea are shown in Figure 1 and Tables S1 and S2. As shown in Figure 1A,B (Table S1), the adjusted ORs for severe dysmenorrhea pain among participants in the highest versus lowest tertiles of dietary indices were not statistically significant. Specifically, the OR (95% CI) was 0.75 (0.26–2.19; *p* = 0.611) for eDII, 0.61 (0.17–2.20; *p* = 0.453) for MEDI‐LITE, and 1.10 (0.42–2.85; *p* = 0.833) for DAL. Similarly, Figure 2 (Table S2) indicated no significant associations between higher adherence to these dietary indices and severe complications of dysmenorrhea. The ORs (95% CI) were 0.67 (0.23–1.95; *p* = 0.469) for eDII, 0.56 (0.15–2.06; *p* = 0.387) for MEDI‐LITE, and 1.44 (0.55–3.76; *p* = 0.450) for DAL.

The findings of ROC curve analysis for evaluating the sensitivity and specificity of dietary indices in differentiating the dysmenorrhea status are indicated in Table 5. The AUC (95% CI) for differentiating the dysmenorrhea status was 0.45 (0.34–0.56), *p*‐value = 0.436 for eDII, 0.41 (0.30–0.52), *p*‐value = 0.124 for MEDI‐LITE, and 0.46 (0.34–0.57), *p*‐value = 0.436 for DAL.

**TABLE 5:** The findings of ROC curve analysis for evaluating the sensitivity and specificity of dietary indices in differentiating the dysmenorrhea status among study participants.

Dietary indices	AUC (95% CI)	*p*	Cut‐points*
eDII	0.45 (0.34–0.56)	0.385	−5.0
MEDI‐LITE	0.41 (0.30–0.52)	0.124	2.0
DAL	0.46 (0.34–0.57)	0.436	−48.08

* Cut‐points with 100% sensitivity and specificity.

Findings indicated that the mentioned dietary indices have a poor ability to differentiate dysmenorrhea status and they are not significant predictors of dysmenorrhea status (as shown in Figure 2). The best points for 100% sensitivity and specificity of differentiating the dysmenorrhea status for eDII, MEDI‐LITE, and DAL were −5.0, 2.0, and −48.08, respectively (as shown in Figure 1A).

## 4 Discussion

In this cross‐sectional study conducted among university students, we investigated the associations between three dietary indices, eDII, MED‐LITE, and DAL, and the odds of primary dysmenorrhea. Our analysis revealed no significant relationship between any of these indices and the occurrence of dysmenorrhea. Furthermore, there was no significant correlation between these indices and the severity of menstrual pain, as measured by VAS. To our knowledge, the current study first assessed the relationship between eDII, MED‐LITE, and DAL indices with the odds of dysmenorrhea.

It is important to note that previous studies have used different instruments to evaluate dietary indices and menstrual pain, making it difficult to compare findings. The results of this study are in line with previous findings reported by Mucuk et al. that showed no significant association between higher adherence to the DII index with primary dysmenorrhea among Turkish university students (Mucuk and Onur 2021). Also, our results follow a previous study among Spanish students that showed the Mediterranean diet wasn't related to menstrual pain and bleeding, although it is inversely related to longer menstrual cycles (Onieva‐Zafra et al. 2020).

The relationship between diet and dysmenorrhea has been investigated previously, with inconsistent results. For example, a randomized controlled trial found that higher fiber and complex carbohydrate intake was associated with reduced dysmenorrhea severity (Kartal and Akyuz 2018a), whereas a study in Indonesia reported no correlation between fiber or snack consumption and dysmenorrhea (Fitrianingsih and Santanu 2021). In our study, participants experiencing menstrual pain reported higher total carbohydrate and fiber intake, reflecting this inconsistency. While causality cannot be established, it is possible that affected individuals consume more fiber‐rich foods in an attempt to alleviate pain (Ciołek et al. 2024), or that higher fiber intake may cause intestinal discomfort that could be mistaken for menstrual pain (Wang et al. 2022; Gonlachanvit et al. 2004). Additionally, some studies have linked higher sugar and sweet consumption to increased dysmenorrhea severity (Alammar et al. 2020), while others found no significant association (Monday et al. 2019).

These contradictions also exist regarding the high consumption of fruits, vegetables, and dairy products for dysmenorrhea. In some studies (Alammar et al. 2020; Barcikowska, Wojcik‐Bilkiewicz, et al. 2020; Bavil et al. 2016), no association between these food groups and dysmenorrhea has been observed, while a systematic review study (Bajalan et al. 2019) reported a protective relation between higher consumption of fruits, vegetables, and milk with dysmenorrhea pain. However, some previous studies have shown that unhealthy eating habits are associated with more experience of dysmenorrhea (Sundari et al. 2020), and several foods such as caffeinated drinks (Monday et al. 2019), ready meals and snack foods (Najafi et al. 2018), consumption of more than 4 glasses of tea and Coca‐Cola per day (Hailemeskel et al. 2016), and low consumption of grains and seafood (Alammar et al. 2020) have been positively associated with dysmenorrhea and its severity. On the other hand, regarding the consumption of meals, at least two studies have reported a protective relationship between daily breakfast consumption and dysmenorrhea (Tadese et al. 2021; Fitrianingsih and Santanu 2021). However, we could not investigate the relationship between breakfast consumption and dysmenorrhea in our study because the consumption of meals is not included in the calculation of any of the eDII, MED‐LITE, and DAL indices. Also, the beneficial effects of various micronutrients such as vitamins D, E, and K and minerals including calcium, magnesium, zinc, and bromine with dysmenorrhea have been reported in studies (Naz et al. 2020; Kartal and Akyuz 2018).

As we examined the diet as a form of inflammatory indices and DAL, it seems that the overall effects of potentially beneficial and detrimental nutritional components related to dysmenorrhea may have been balanced out by their interactions within the indices; this could have resulted in the non‐significant association between the investigated indices and dysmenorrhea observed in our study.

The observed discrepancies between the findings of various studies can likely be attributed to differences in study design, sample size, eating habits and patterns among communities, methods of measuring food intake, dysmenorrhea symptoms, and complications evaluation methods, and the lack of proper distinction between primary and secondary dysmenorrhea in studies. On the other hand, it appears that since dysmenorrhea is a brief and short‐term event caused by menstruation and typically lasts only a few days, nutritional evaluations during the time of dysmenorrhea pain and slightly before that are much more effective than the evaluation of annual food intake and general diet indicators. Consequently, this could also contribute to the lack of statistical significance between our dietary indices and dysmenorrhea in the current study.

Certain herbal beverages, such as chamomile (Niazi and Moradi 2021) and fennel (Shahrahmani et al. 2021), have demonstrated beneficial effects on dysmenorrhea pain in previous systematic reviews. The impact of a person's diet during dysmenorrhea may be more significant than the diet they follow in the last month or previous year. Consequently, it highlights the potential for thoughtful food choices to aid in managing dysmenorrhea.

Our study possesses several strengths. Firstly, we investigated the association of various dietary indices including the eDII, MED‐LITE, and DAL, with the odds of dysmenorrhea among university students. Dietary intakes were assessed by trained dietitians through face‐to‐face interviews, employing a validated FFQ. Furthermore, diverse analytical approaches, including correlation analysis, multivariable regression, and ROC curve analysis, were employed to examine potential associations. This study has several limitations that warrant consideration. Its cross‐sectional design precludes any inference of causality, and the relatively small sample size may have limited the statistical power to detect modest associations. Although the use of a validated FFQ likely reduced measurement error in estimating dietary intake, inherent limitations such as recall bias remain. Furthermore, because dysmenorrhea is an episodic condition, annual dietary assessments may not adequately reflect short‐term dietary variations or premenstrual dietary patterns that could influence pain severity. In addition, due to cultural and religious factors leading to minimal and underreported alcohol consumption, we excluded this component from the MEDI‐LITE score, which may affect its validity and comparability with other non‐Iranian studies. Even after adjusting for some potential confounders, the possibility of unmeasured and unknown residual confounders may have contributed to confounding effects. Furthermore, since our study population was limited to university students, the lack of significant findings may be partly attributed to the potential homogeneity in their dietary intake. Additionally, the generalizability of our results to the broader female population, particularly women receiving clinical treatment for dysmenorrhea, may be limited. Future research should employ prospective designs, tracking dietary intake and menstrual symptoms over multiple cycles, to clarify temporal relationships. Daily food diaries or repeated 24‐h recalls during the premenstrual and menstrual phases may provide more sensitive data than FFQ.

In conclusion, the present study did not reveal any significant associations between dietary indices, including eDII, MED‐LITE, and DAL, and primary dysmenorrhea. Further research is needed to clarify this relationship and to identify other potential dietary risk factors for primary dysmenorrhea. We suggest that future studies also focus on the relationship between food intake during the time of dysmenorrhea pain and slightly before that using detailed food record questionnaires or 24‐h recall methods and the symptoms of dysmenorrhea.

## Author Contributions

**Mina Salek:** conceptualization (equal), data curation (equal), formal analysis (equal), methodology (equal), writing – original draft (equal). **Farshad Teymoori:** formal analysis (equal), methodology (equal), validation (equal), writing – review and editing (equal). **Ladan Haghighi:** investigation (equal), writing – review and editing (equal). **Masoud Salehi:** formal analysis (equal), methodology (equal). **Saeed Tavakkoli:** data curation (equal), writing – original draft (equal). **Minoo HasanRashedi:** investigation (equal), writing – review and editing (equal). **Farzad Shidfar:** supervision (equal), validation (equal), writing – review and editing (equal).

## Funding

This study was financially supported by the Iran University of Medical Sciences, Tehran, Iran.

## Ethics Statement

The study protocol was approved by the ethics research council of the Research Institute for Iran University of Medical Sciences.

## Consent

Written informed consents were obtained from all participants.

## Conflicts of Interest

The authors declare no conflicts of interest.

## Supporting information


**Supplementary Table 1:** The odds ratio and 95% confidence interval of severe pain of dysmenorrhea across tertiles of dietary indices among study participants.
**Supplementary Table 2:** The odds ratio and 95% confidence interval of severe complications of dysmenorrhea across tertiles of dietary indices among study participants.

## Data Availability

The datasets used and analyzed during the current study are available from the corresponding author upon reasonable request.
