# Supplementary material for: Dietary Acid Load, Empirical Dietary Inflammatory Index, and Literature‐Based Adherence to Mediterranean Diet Score Relationship With Primary Dysmenorrhea
Source: Food Sci Nutr. 2026 Apr 27;14(5):e71580. doi: 10.1002/fsn3.71580 (PMC13121908; doi:10.1002/fsn3.71580)
Supplement: Supplementary file 1 — Supplementary Table 1: The odds ratio and 95% confidence interval of severe pain of dysmenorrhea across tertiles of dietary indices among study participants. Supplementary Table 2: The odds ratio and 95% confidence interval of severe complications of dysmenorrhea across tertiles of dietary indices among study participants. [file FSN3-14-e71580-s001.docx]

**Supplementary Data**

**Supplementary Table 1: The odds ratio and 95% confidence interval of** **severe pain of dysmenorrhea across tertiles of dietary indices among study participants**

|  | **Tertile 1** | **Tertile 2** | | **Tertile 3** | |
| --- | --- | --- | --- | --- | --- |
| **Predictor Variables** | **Ref** | **OR (95%CI)** | **P-value** | **OR (95%CI)** | **P-value** |
| **eDII** |  |  |  |  |  |
| Crude model | **Ref** | 0.60 (0.28 – 1.57) | 0.358 | 0.63 (0.23 – 1.70) | 0.367 |
| Adjusted model | **Ref** | 0.72 (0.29 – 1.75) | 0.471 | 0.75 (0.26 – 2.19) | 0.611 |
| **MEDI-LITE** |  |  |  |  |  |
| Crude model | **Ref** | 0.52 (0.24 – 1.14) | 0.104 | 0.52 (0.15 – 1.77) | 0.303 |
| Adjusted model | **Ref** | 0.56 (0.25 – 1.25) | 0.164 | 0.61 (0.17 – 2.20) | 0.453 |
| **DAL (mEq/d)** |  |  |  |  |  |
| Crude model | **Ref** | 1.53 (0.63 – 1.68) | 0.340 | 0.91 (0.37 – 2.23) | 0.849 |
| Adjusted model | **Ref** | 1.52 (0.60 – 3.85) | 0.369 | 1.10 (0.42 – 2.85) | 0.833 |

DAL; Dietary acid load, eDII; Empirical dietary inflammatory index, MEDI-LITE; Literature-Based Adherence to Mediterranean diet Score.

Analyses were adjusted for age, menarche age, education levels, physical activity, BMI, and energy intake

**Supplementary Table 2: The odds ratio and 95% confidence interval of severe complications of dysmenorrhea across tertiles of dietary indices among study participants**

|  | **Tertile 1** | **Tertile 2** | | **Tertile 3** | |
| --- | --- | --- | --- | --- | --- |
| **Predictor Variables** | **Ref** | **OR (95%CI)** | **P-value** | **OR (95%CI)** | **P-value** |
| **eDII** |  |  |  |  |  |
| Crude model | **Ref** | 0.74 (0.32 – 1.74) | 0.498 | 0.61 (0.22 – 1.64) | 0.331 |
| Adjusted model | **Ref** | 0.81 (0.33 – 1.98) | 0.653 | 0.67 (0.23 – 1.95) | 0.469 |
| **MEDI-LITE** |  |  |  |  |  |
| Crude model | **Ref** | 0.52 (0.24 – 1.14) | 0.105 | 0.40 (0.11 – 1.39) | 0.151 |
| Adjusted model | **Ref** | 0.53 (0.24 – 1.19) | 0.130 | 0.56 (0.15 – 2.06) | 0.387 |
| **DAL (mEq/d)** |  |  |  |  |  |
| Crude model | **Ref** | 1.87 (0.77 – 4.52) | 0.164 | 1.16 (0.47 – 2.82) | 0.737 |
| Adjusted model | **Ref** | 1.73 (0.68 – 4.39) | 0.247 | 1.44 (0.55 – 3.76) | 0.450 |

DAL; Dietary acid load, eDII; Empirical dietary inflammatory index, MEDI-LITE; Literature-Based Adherence to Mediterranean diet Score.

Analyses were adjusted for age, menarche age, education levels, physical activity, BMI, and energy intake
